# Self-study and online interactive case-based discussion to improve knowledge of medical students in the COVID-19 era

**DOI:** 10.1186/s12909-024-05578-w

**Published:** 2024-05-25

**Authors:** Maliwan Oofuvong, Sumidtra Prathep, Prae Plansangkate, Jutarat Tanasansuttiporn, Chutida Sungworawongpana, Wilasinee Jitpakdee

**Affiliations:** Department of Anesthesiology, Faculty of Medicine, Prince of Songkla University, 15 Kanjanavanich Road, Songkhla, 90110 Thailand

**Keywords:** Self-study, Online interactive case-based discussion, Knowledge domain, Medical students, COVID-19 era

## Abstract

**Background:**

We aimed to determine whether a new online interactive learning method for fifth-year medical students could improve their knowledge of pre- and postoperative care during the COVID-19 era.

**Methods:**

A retrospective cohort study was conducted from June 2020 to May 2022 during the pre- and postoperative care course for fifth-year medical students in a university hospital in southern Thailand. Students in the 2020 cohort received only a 60-minute lecture on spinal anesthesia via Zoom while a 3-step online interactive learning method was used for the 2021 cohort. Step 1: students performed self-study comprised of video lectures and case-based discussion one week before the online class with a pre-test submitted via Google forms. Step 2: an online interactive case-based discussion class was performed via Zoom by two experienced anesthesia staff and a post-test was submitted by the students via Google forms. Step 3: a small group discussion of course evaluation between 13 representatives of students and anesthesia staff was performed via Zoom. A comparison of the post-test and pre-test scores containing 20 multiple choice questions as well as the final exam scores before (2020) and after (2021) the new interactive learning was performed using a *t*-test.

**Results:**

There were 136 and 117 students in the 2020 and 2021 academic years, respectively. The final mean (SD) exam scores for the 2020 and 2021 academic years were 70.3 (8.4) and 72.5 (9.0), respectively with a mean (95% confidence interval (CI)) difference of 2.2 (4.3, -0.02). In 2021, the mean (95% CI) difference between the post-test and pre-test scores was 5.8 (5.1, 6.5). The student representatives were satisfied with the new learning method and gave insightful comments, which were subsequently implemented in the 2022 academic year course.

**Conclusion:**

The new interactive learning method improved the knowledge of fifth-year medical students attending pre- and postoperative care course during the COVID-19 era. The final exam scores may not be suitable to represent the overall outcomes of the new interactive learning method. Using an online two-way communication method can improve the overall satisfaction and course adaptation during the COVID-19 era.

**Supplementary Information:**

The online version contains supplementary material available at 10.1186/s12909-024-05578-w.

## Background

Since the COVID-19 pandemic, disruptions in medical education were unavoidable. Adaptations of the medical education system among health care learners were developed [1], including long distance or online learning, telemedicine, e-learning [2], online video lecture [3], and online synchronous live streaming sessions [4]. Niriella et al. [1] suggested that a medical education program during the COVID-19 pandemic should be flexible and involve collaboration between learners and facilitators. Due to the COVID-19 pandemic face-to-face lectures in 2020 were forcibly changed to online lectures via Zoom the following year. However, online teaching programs encounter less interaction and active participation between learners and teachers [5] and hands-on skills and real-life experience, which are required for undergraduate medical students, are limited [5]. In 2021, we implemented a new and interactive online learning method to improve student’s knowledge of patient care. From a literature review, the outcomes of online learning during the COVID-19 pandemic were satisfaction and perception of the learning method [4, 6–8]. Therefore, we aimed to assess the usefulness of this new teaching method at the end of the semester.

## Materials and methods

This retrospective cohort study was conducted after approval was granted by the Ethics Committee, Faculty of Medicine, Prince of Songkla University on January 28, 2022 (REC 65-047-08-1). The inform consent was waived by the Human Research Ethics Committee of the Faculty of Medicine, Prince of Songkla University, due to retrospective nature of the study. We recruited all fifth-year medical residents in the 2020 and 2021 academic years who attended the final exam in our pre- and postoperative care course at the Faculty of Medicine, Prince of Songkla University. Students who did not attend the final exam were excluded.

### Standard Operating Procedure for pre- and postoperative care course

#### 2020 academic year

During the COVID-19 pandemic, face-to-face lecture was changed to online lecture. Students can access through the 10-MCQs quiz by e-learning: Case Based Spinal Anesthesia from PSU website (URL:https://lms2.psu.ac.th/course/view.php? id=8064) voluntarily before the lecture day. On the lecture day, an online 60-minute lecture via Zoom by single staff was performed.

#### 2021 Academic year (Fig. 1)

**Fig. 1:**
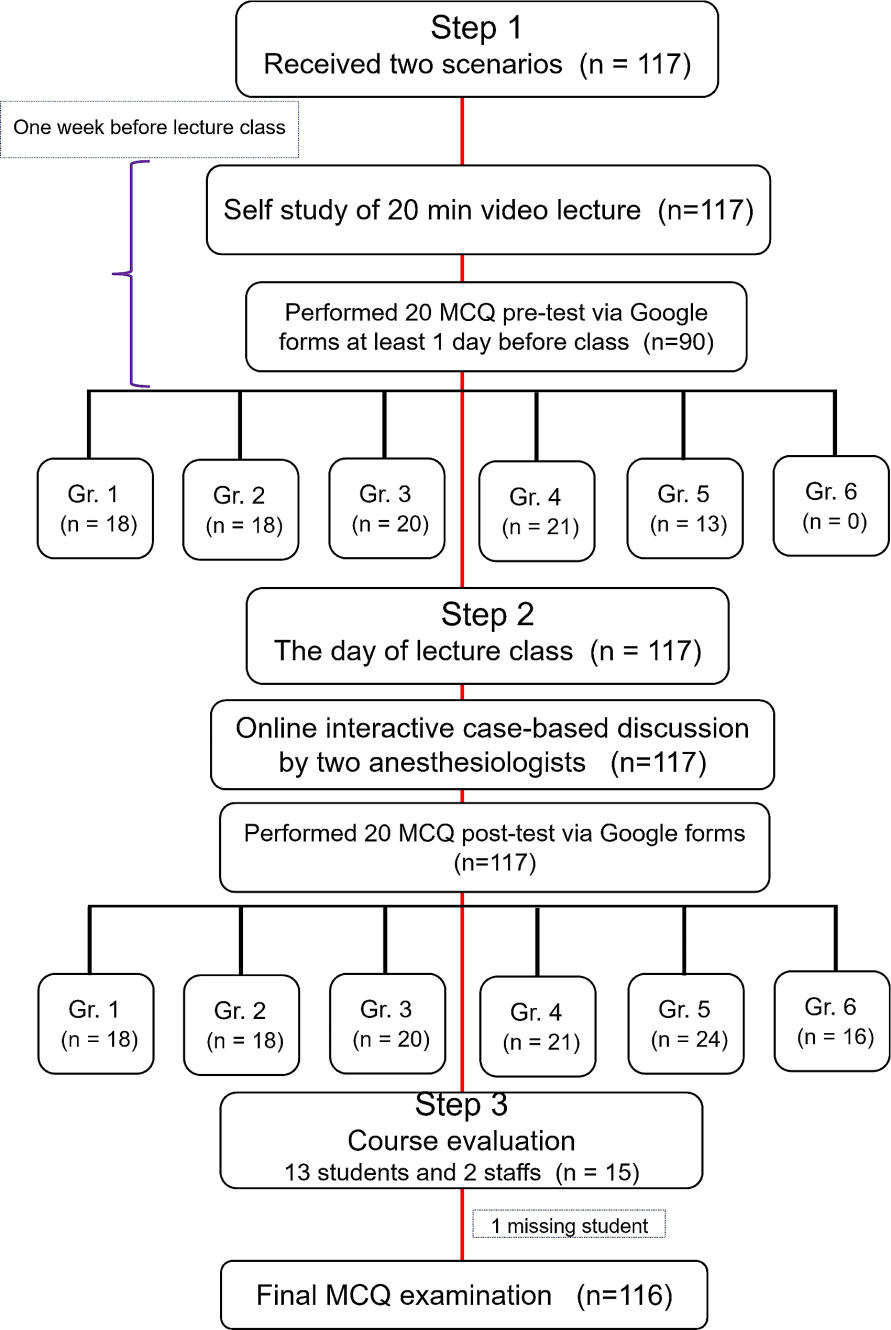
Flow diagram of the study *Gr*. group, *MCQs* multiple choice questions

Six rotations consisting of fifth-year medical students attended interactive case-based discussion sessions from May to July 2021 and each rotation was scheduled every 2 weeks. There were 3 steps contained in the new learning method. Step 1: students performed self-study comprising video lectures and case-based discussion sessions held one week before the online class with a pre-test submitted via Google forms. Step 2: an online interactive case-based discussion session was held via Zoom by two experienced anesthesia staff and a post-test was submitted by the students via Google forms. Step 3: 13 student representatives, together with anesthesia staff, held a discussion session via Zoom to evaluate the course. The student representatives consisted of a president and two representatives from each group (12 students). They were all active participants who were willing to discuss the advantages and disadvantages of the course.

### Outcome of the study and outcome measurement

The two outcomes of the study were the final exam scores from the pre- and postoperative care course given before (2020) and after (2021) the new interactive learning method. The difference in the scores between post-test and pre-test of the Spinal Anesthesia Section was also compared. The students in the 2021 academic year were divided into a six-group rotation. The post-test scores among groups were also compared since they were aware of the post-test.

#### Development and validation of questionnaires

Two Zoom meetings, held in May and June 2021, were supervised by eight teaching staff among the six rotations. After the first two rotations, four of the 20 MCQs were simplified. Therefore, the questions from the 4th to 6th rotations were the same but slightly different from the 1st and 2nd rotation. However, the pre-test and post-test questionnaires were the same throughout the six rotations (additional file 1).

### Potential predictors of the final exam score

Potential predictors of the final exam score in the 2021 academic year were age, sex, rotation, pre-test score, post-test score, and performing extra self-learning such as e-learning, computer-assisted instruction, or other methods.

### Sample size calculation

For the main objective, we hypothesized that the final score from the 2021 academic year would be 7 points (10% increase) higher than the score from the 2020 academic year with a standard deviation of 15 points under a level of significance of 0.05 and 80% power to detect this increase. Thus, the required sample size, assuming a 10% drop out rate from the final year, was 108 students. For the secondary objectives, we hypothesized that the post-test score among groups would differ by 2.5 points (out of 20) with a standard deviation of 2 points under a level of significance of 0.05 and 80% power to detect the difference. Thus, the required sample size was 13 residents per group with compensation of a 10% drop out rate. Therefore, a total of 120–130 students each year was deemed to be adequate.

### Statistical analysis

Continuous variables were presented using the median and interquartile range for non-normally distributed data and the mean and standard deviation for normally distributed data. Categorical variables were presented using frequency and percentage and compared using Fisher’s exact test or Pearson’s Chi-square test. Continuous variables were compared using Kruskal-Wallis tests and analysis of variance when comparing more than two groups. For two group comparisons, continuous variables were compared using Student’s t-test and the Wilcoxon rank-sum test. Predictors associated with exam scores were compared using a multivariate linear regression model using a stepwise backward elimination method to select the best model. We included all exploratory variables into the initial multivariate linear regression model regardless of their statistical significance from the univariate analysis since they were all related to internal validity threat. The strengths of the associations were presented as beta coefficients with 95% confidence limits.

## Results

There were 136 and 117 students in the 2020 and 2021 academic years, respectively. The process of the new online interactive learning method in the 2021 academic year is shown in Fig. 1. Ninety students from rotations 1–5 performed the pre-test while 117 from all six rotations performed the post-test. In step 3, all students were satisfied with the new learning method. The self-study 20-minute video lecture was reported to be concise and relevant with the learning objectives. The case-based discussion represented the real case situation in the operating theater. The length of the post-test was appropriate and covered all the learning objectives. The student representatives suggested to include the reports of the post-test score and the key answers of the MCQs in the post-test Google forms. These suggestions were developed and included in the 2022 academic year.

**Fig. 2:**
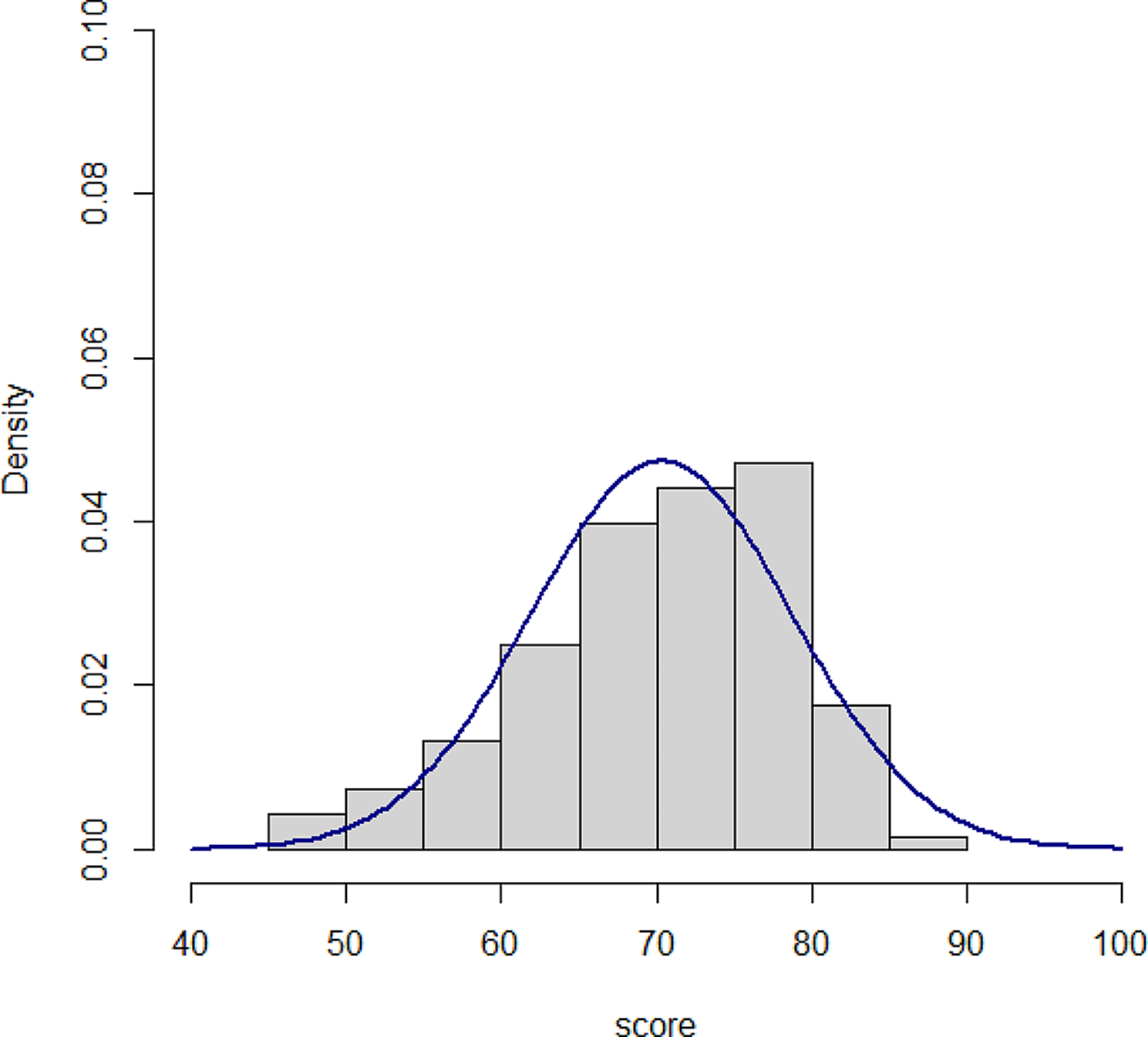
Histogram of final exam scores for the 2020 academic year

**Fig. 3:**
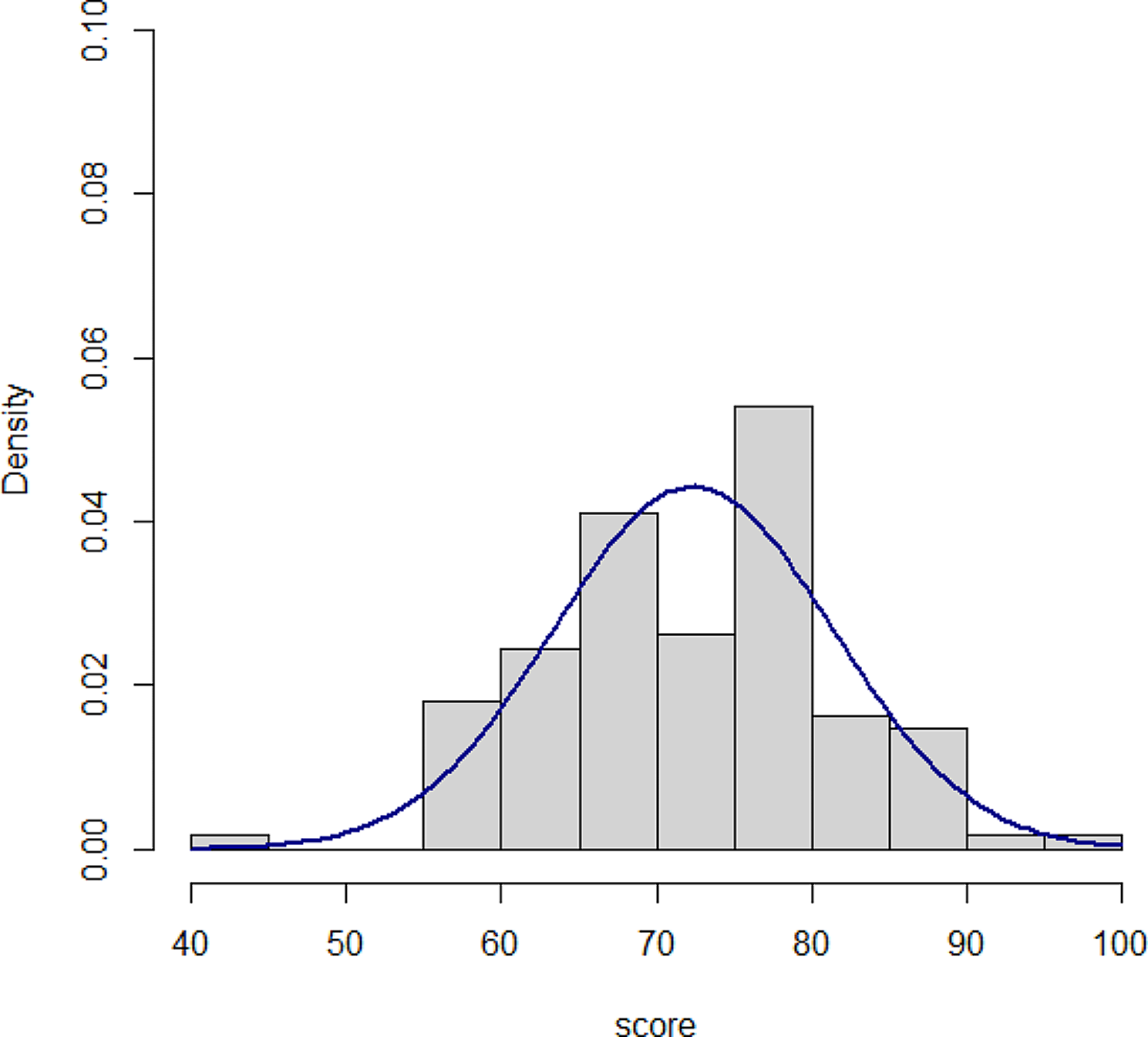
Histogram of final exam scores for the 2021 academic year

Characteristics of the medical students among the six rotations in the 2021 academic year are shown in Table 1. There were no differences between the six groups in terms of age and sex. Students in the 6th rotation performed more e-learning (*p* < 0.031) and other self-learning (*p* < 0.001) tasks than those in the other rotations; however, self-study by computer assisted instruction and video methods were no different. Pre-test and post-test scores among the six rotations in the 2021 academic year are shown in Table 2. The post-test scores were significantly different among the six rotations (*p* = 0.002) but not the difference between pre- and post-test scores (*p* = 0.325). Pre-test score, post-test score and the difference in pre-test and post-test scores for students in the 2021 academic year are shown in Table 3. Overall, the post-test score was significantly higher than those in pre-test score (mean [95% confidence interval]: 5.81 [5.07, 6.54], *p* < 0.001). The final exam scores for students in both academic years are also shown in Table 3. Students in the 2021 academic year had a higher final exam score compared to those in the 2020 academic year (70.3 vs. 72.5, *p* = 0.05). Figures 2 and 3 show the histogram of final MCQs score in 2020 and 2021 academic year, respectively which represent normal distribution.

**Table 1:** Comparison of characteristics between medical students in six rotations in the 2021 academic year (*N* = 117)

Characteristic	Group 1 (*n* = 18)	Group 2 (*n* = 18)	Group 3 (*n* = 20)	Group 4 (*n* = 21)	Group 5 (*n* = 24)	Group 6 (*n* = 16)	*p* value
Sex (F/M)	11/7	9/9	12/8	10/11	13/11	9/7	0.952
Age (years), median (IQR)	22 (22, 22)	22 (22, 23)	22 (22, 23)	22 (22, 23)	23 (22, 23)	22 (22, 23)	0.247^+^
Self-study by CAI	8 (44.4)	9 (50)	7 (35)	8 (38.1)	6 (25)	9 (56.2)	0.399
Self-study by e-learning	15 (83.3)	13 (72.2)	11 (55)	16 (76.2)	16 (66.7)	16 (100)	0.031*
Previous video self-learning	16 (88.9)	17 (94.4)	18 (90)	21 (100)	24 (100)	16 (100)	0.206
Other self-learning methods	10 (55.6)	4 (22.2)	9 (45)	4 (19)	7 (29.2)	13 (81.2)	< 0.001 **

*Note *Data are presented as frequency (%) and mean (standard deviation) unless stated otherwise. ^+^ Kruskal-Wallis test, * Fisher’s exact test, ** Chi square test. *CAI* computer-assisted instruction, *IQR* interquartile range

**Table 2:** Pre-test and post-test scores among six rotations in the 2021 academic year (*N* = 117)

Score	Group 1 (*n* = 18)	Group 2 (*n* = 18)	Group 3 (*n* = 20)	Group 4 (*n* = 21)	Group 5 (*n* = 24)	Group 6 (*n* = 16)	*p* value
Pre-test	8 (6, 10)	8 (6.2, 12)	11.5 (7.8, 13.2)	9 (7, 9)	9.2 (7.8, 9.4)^+^	NA^++^	0.138
Post-test	13 (12, 13.8)	13.5 (12.2, 15.8)	17 (14.5, 20)	15 (11, 18)	16.5 (14.5, 19)	17 (13, 19)	0.002*
Post-test – pre-test: Mean (SD)	4.3 (3.5)	4.9 (4.8)	5.8 (5)	6.1 (3.2)	6.6 (3.8)	7 (3)	0.325

*Note *Data presented as median (IQR) unless stated otherwise, ^+^*n* = 13, ^++^*n* = 0, * Kruskal-Wallis test

**Table 3:** Pre-test, post-test, and final exam scores for the 2020 and 2021 academic years

	2020 (*n* = 136)	2021 (*n* = 117)	*p* value*
Pre-test	-	9.21 (3.05)	-
Post-test	-	15.03 (3.45)	-
Post-test – pre-test, mean (95% CI)	-	5.81 (5.07, 6.54)	< 0.001
Final MCQs exam	70.3 (8.4)	72.5 (9)^+^	0.051
Difference in final exam, mean (95% CI)	2.2 (4.31, -0.02)		

*Note *Data presented as mean (SD) unless stated otherwise. ^+^*n* = 116, * Student’s t-test test. *SD* standard deviation, *CI* confidence interval, *MCQ* multiple choice question

### Predictors of final exam score

Significant predictors of the final exam score in the 2021 academic year are shown in Table 4. After adjusting for rotation and pre-test score, age (*p* = 0.043), and performing extra self-learning (*p* = 0.019) were significant predictors based on the multivariate regression model.

**Table 4:** Potential predictors for final exam scores among medical students in the 2021 academic year (*n* = 89)

Predictor	Crude β (95% CI)	*p* value**	Adjusted β (95% CI)	*p* value*	*p* value**
Age (years)	-1.51 (-3.23, 0.2)	0.084	-2.17 (-4.27, -0.07)	0.043	0.043
Rotation (ref = group 1)		0.021			0.087
Group 2	-0.06 (-5.83, 5.72)		1.27 (-4.29, 6.83)	0.652	
Group 3	6.68 (1.13, 12.24)		6.62 (1.25, 11.99)	0.016	
Group 4	1.85 (-3.64, 7.34)		4.88 (-0.66, 10.43)	0.083	
Group 5	-1.24 (-6.56, 4.09)		1.62 (-4.52, 7.76)	0.601	
Pre-test score	0.45 (-0.07, 0.98)	0.087	0.44 (-0.08, 0.96)	0.098	0.098
Extra self-learning	1.00 (-2.43, 4.44)	0.562	4.86 (0.81, 8.91)	0.019	0.019
r^2^ of final model	0.14				

Note *t-test, **F-test. One student in the pre-test did not attend the final exam. *β* beta coefficients, *CI* confidence interval. Group 6 was omitted in the model since they did not complete the pre-test

## Discussion

Our new interactive learning method in the 2021 academic year improved the final exam scores by two points with marginal significance. Post-test scores improved significantly after the new intervention compared to the pre-test score. The new interactive learning method comprised of the combination of self-study and online interactive case-based discussion, which was similar to the blended learning approach reported by other studies [9, 10]. The Blended method helped to stimulate active learning attitudes and improve clinical practice among medical students. However, blended learning involves the integration of online and face-to-face learning, the latter method difficult to implement during the COVID-19 pandemic. Hence, our online interactive case-based discussion was another way to stimulate active learning attitudes in this circumstance in order to facilitate critical, creative, and complex thinking skills [9].

The final exam score improved marginally, which could arise from the main cause that the anesthesia content was a part of the whole content of the final exam of the pre- and post-operative care course and other factors such as the more appropriate study design, the possible internal validity threat [11, 12], or the appropriate outcome measurement may have contributed. Since the COVID-19 pandemic was an unpredictably emerging infectious disease, the new online interactive learning was promptly managed to promote the continuity of medical education learning among undergraduate students. A prospective intervention trial could not be applied in this circumstance. In order to minimize the internal validity threat, we tried to determine the potential predictors of the final exam score related to the confounders. The extra self-learning was one of the important predictors which could gain almost a 5-point increment compared to the one without extra self-learning. The extra self-learning combination with the new online interactive learning could improve final exam scores during the COVID-19 pandemic. A systematic review published in 2010 reported that self-directed learning (SDL) in health professions education was associated with moderate improvement in the knowledge domain compared with traditional teaching methods [13]. Thota et al. [14] also supported our finding that lecture cum method which included interactive discussion combination with SDL session improved the post-test score in biochemistry students. We also found the older age was related with lower final exam score. The age range of our 5th year medical student was ranged from 21 to 28 years old. From the multivariate model, a one-year increase in age decreased the score by 2 points. Many studies reported that the learning process may decline with age especially in implicit probabilistic sequence learning and memory performance [15–17]. Finally, comparison of final exam scores between the 2020 and 2021 academic years may be hampered for the following reasons. First, students in these two years may have different SDL styles; some students in the 2020 cohort may have attended the 10-MCQs quiz via e-learning (pretest). Second, they were different groups of students to compare in different years, thus the knowledge gap may be different. Third, contents of the exams differed depending on the specific objectives. Therefore, to evaluate the fifth-year medical student’s performance, the process evaluation to measure the skills performance, such as procedure practice with manikin or with real patients in the operating theater, or use of Objective Structured Clinical Examination simultaneously with MCQ, Short Answer Questions, or Key Features Test, as well as their satisfaction, should be taken into account.

### Implications of the study

Our new online interactive learning method significantly improved post-test scores, and marginally improved final exam scores. Using an online two-way communication method can improve the overall satisfaction among students and facilitators. Nonetheless, the faculty should make sure that there are no poor information technology skills or lack of internet facility issues among learners and facilitators to greatly facilitate online learning process [18].

### Strengths and limitations of the study

The strengths of our study are the adequate sample size to examine the main outcome and the performance of the multivariate linear regression to find potential predictors for the final exam score. However, there are some limitations of this study. First, the nature of the retrospective cohort study could encounter with some information bias such as characteristic of students in the 2020 academic year. Second, there were some missing data in the pre-test scores since the announcement of the online pre-test course was phased down at the end of the semester. However, the pre-test score had no significant impact on the final exam according to the final multivariate model. The generalizability of our results is limited to single university hospitals but can be generalized to other universities in the same setting.

## Conclusions

A new interactive learning method improved the knowledge of fifth-year medical students attending the pre- and postoperative care course during the COVID-19 era. However, the final exam scores may not entirely represent the overall outcomes of the new interactive learning method. Using an online two-way communication method can improve the overall satisfaction and course adaptation during the COVID-19 era.

**Supplemental file**.

### Electronic supplementary material

Below is the link to the electronic supplementary material.

Supplementary Material 1

## Data Availability

The datasets used and/or analyzed during the current study are available from the corresponding author on reasonable request.

## Abbreviations

*MCQs*: Multiple choice questions
*SDL*: Self-directed learning
