# Supplementary material for: Self-study and online interactive case-based discussion to improve knowledge of medical students in the COVID-19 era
Source: BMC Med Educ. 2024 May 25;24:576. doi: 10.1186/s12909-024-05578-w (PMC11128110; doi:10.1186/s12909-024-05578-w)
Supplement: Supplementary file 1 — Supplementary Material 1 [file 12909_2024_5578_MOESM1_ESM.pdf]

Pre-test and post-test quiz, version 1

1. What is the spinal anesthesia also called?
A. Dura block
B. Epidural block
C. Subdural block
D. Intrathecal block

2. What type of operation is appropriate for spinal anesthesia?
A. Anal fissure
B. Chest wall surgery
C. Laparoscopic herniorrhaphy
D. L4-5 Laminectomy

3. What is the absolute contraindication of spinal anesthesia?
   A. Patient’s refusal
   B. Back pain
   C. Previous spine surgery
   D. Cardiac disease

4. What is the appropriate sensory dermatome for cesarean section under spinal anesthesia?
   A. level of nipples
   B. level of xiphoid process
   C. level of umbilicus
   D. inguinal or groin region

5. Which is the most common level of spinal cord ending in adults?
   A. T12
   B. L1
   C. L3

D. S2

6. What is correct ordering regarding differential blockade of local anesthetics from cephalad to caudad?
   A. sensory blockade, sympathetic blockade and motor blockade
   B. motor blockade, sympathetic blockade and sensory blockade
   C. sympathetic blockade, sensory blockade and motor blockade
   D. sympathetic blockade, motor blockade and sensory blockade

7. Which layer can we feel “loss of resistance” when performing spinal anesthesia before reaching the subarachnoid space?
   A. supraspinous ligament
   B. interspinous ligament
   C. ligamentum flavum
   D. epidural space

8. A patient who had inactive asthma as an underlying disease came for knee replacement surgery. He felt chest discomfort 15 minutes after spinal anesthesia. What is the most likely cause of this discomfort?
   A. Phrenic nerve paralysis
   B. Proprioception block
   C. Inhibition of cardiac accelerator fiber
   D. Sympathetic block

9. What should the anesthesiologist be most concerned with during spinal anesthesia?
   A. Sterile technique
   B. Patient education
   C. Obtaining written informed consent
   D. Following the standard operating procedures

10. Which of the following is correct in order to minimize the risk of a post-dural puncture headache?

A. Use of 27G spinal needle

B. Use of Quincke spinal needle

C. Place the spinal needle bevel perpendicular to the spine level

D. Lateral position during spinal anesthesia

11. A 60-year-old man came for a thrombosed hemorrhoidectomy with prone position. He had an abrasion wound grade 2 with redness at the lower spine. He had no fever. What should be the next management in this case?

A. Postpone surgery

B. Spinal anesthesia

C. General anesthesia

D. Local anesthesia

12. What is the appropriate sensory dermatome for inguinal hernia repair under spinal anesthesia?

A. Nipple

B. Xyphoid

C. Umbilicus

D. Groin

13. What is the appropriate preparation for cesarean section under spinal anesthesia?

A. Stand-by propofol

B. Stand-by dopamine

C. Perform maximum barrier

D. Prepare 20G spinal needle

14. The sensory dermatome level of T4 was tested 10 minutes after spinal anesthesia for cesarean section. Blood pressure was 70/40 mmHg with no bradycardia. What is the most common cause of hypotension in this case?

A. Proprioception block

B. Inhibition of cardiac accelerator fiber

C. Sympathetic block

D. Parasympathetic block

15. The sensory dermatome level of T6 was tested 10 minutes after spinal anesthesia. Blood pressure was 80/50 mmHg. What should be the next management?

A. Check sensory level

B. Check consciousness

C. Fluid loading

D. Dopamine infusion

16. What is the possible reason to encourage absolute bed rest for at least 4 hours after spinal anesthesia?

A. Loss of proprioception

B. Residual sympathetic block

C. Prevent postural hypotension

D. All of the above

17. What is the supportive treatment of post-dural puncture headache?

A. Nil per oral

B. Early ambulation

C. Paracetamol around the clock

D. Epidural blood patch

18. A patient developed vomiting 20 minutes after spinal anesthesia. The maximum sensory dermatome level was T10. The blood pressure was 110/70 mmHg. What is the most common cause of vomiting in this patient?

A. Cerebral hypoperfusion

B. Sympathetic block

C. Prominence of parasympathetic function

D. Dehydration

19. What is a possible cause of urinary retention after spinal anesthesia?

A. Intrathecal morphine

B. Sympathetic block

C. Parasympathetic block

D. All of the above

20. What is the appropriate test/procedure to minimize the risk of post-dural hypotension after spinal anesthesia?

A. The difference in blood pressure during position change does not exceed 20 mmHg.

B. Absolute bed rest for at least 6 hours.

C. Sensory dermatome level below L1.

D. All toes can move spontaneously.
